# Poly‐IC Alleviates Nitroglycerin‐Induced Migraine by Inhibiting Neuroinflammation via TLR3/TRIF Signaling Pathway

**DOI:** 10.1111/cns.70444

**Published:** 2025-05-23

**Authors:** Ye Hong, Mengmeng Ma, Changling Li, Yang Zhang, Yanbo Li, Ning Chen, Jinghuan Fang, Li He

**Affiliations:** ^1^ Department of Neurology West China Hospital of Sichuan University Chengdu China; ^2^ Department of Neurology The Third Affiliated Hospital of Sun Yat‐Sen University Guangzhou China

**Keywords:** migraine, neuroinflammation, neurons, poly‐IC, TLR3/TRIF signaling way

## Abstract

**Aims:**

Toll‐like receptors (TLRs) play critical roles in pain modulation and immune responses. Polyinosinic‐polycytidylic acid (Poly‐IC), a TLR3‐specific ligand, has shown promise in exerting neuroprotective effects, as it mitigates inflammation in several diseases. Considering that sterile neurogenic inflammation is involved in the pathogenesis of migraine, we explored the impact of Poly‐IC on episodic migraine treatment and the potential mechanisms involved.

**Methods:**

Episodic migraine was induced in male rats via a single intraperitoneal injection of nitroglycerin. Poly‐IC (with or without a TLR3 inhibitor) treatment was performed before migraine induction. Pain was assessed according to the mechanical sensitivity threshold, head‐directed grooming, and the Rat Grimace Scale. The expression of TLR3 and its downstream molecule TRIF was subsequently examined, after which calcitonin gene‐related peptide (CGRP), c‐fos, and proinflammatory cytokine expression was assessed. Moreover, TRIF expression in primary cultured neurons was knocked down by shRNA in vitro to further explore the mechanisms by which Poly‐IC mediates migraine‐like inflammation.

**Results:**

Poly‐IC treatment significantly upregulated TLR3/TRIF expression, reduced the production of CGRP, c‐fos, and inflammatory cytokines, and alleviated allodynia in an animal model of migraine. Moreover, TRIF knockdown blunted the anti‐inflammatory effects of Poly‐IC treatment in primary cultured neurons.

**Conclusions:**

Poly‐IC exerts therapeutic effects against neurogenic inflammation via the TLR3/TRIF signaling pathway in an episodic migraine model.

## Graphical abstract

A promising new drug target for the migraine treatment: Poly‐IC delivery can activate TLR3/TRIF signaling pathway and exert protective effects by blocking pro‐inflammatory responses in migraine.

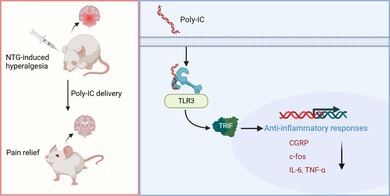

## 1 Introduction

Migraine is a common and debilitating neurological disorder characterized by recurring and unpredictable episodic attacks of intense head pain that affects 15% of the population worldwide [1, 2]. However, the current therapeutic strategies are not completely effective due to the limited options available [3]. Moreover, poor treatment efficacy is observed among individuals with episodic migraine; this leads to longer periods of exposure to pain and increases the risk of disabling chronic migraine, which causes a considerable economic burden to society [4, 5]. Consequently, novel treatment options for migraine are urgently needed.

A series of studies has suggested that sterile neurogenic inflammation is involved in the pathogenesis of migraine [6, 7]. The activation of the trigeminovascular system releases several neuropeptides and cytokines that participate in nociceptive transmission [8, 9]. Calcitonin gene‐related peptide (CGRP) is a critical neuropeptide that is pathologically released from primary afferent neurons and that acts as a nociceptive mediator to accelerate inflammatory conditions [10, 11, 12, 13]. Thus, regulation of inflammatory mediators may provide a therapeutic advantage for migraine treatment.

Endosomal toll‐like receptor 3 (TLR3) is an innate immune receptor that is widely expressed in the central nervous system (CNS) and can be activated upon inflammatory stimulation in response to pathogens [14]. Upon activation, TLR3 recruits the regulatory protein Toll/IL‐1 receptor domain‐containing adapter, which induces IFN‐β (TRIF) to participate in inflammatory activities [15, 16]. Polyinosinic‐polycytidylic acid (Poly‐IC), a TLR3‐specific ligand, has shown promise in exerting neuroprotective effects, as it mitigates inflammatory actions in several diseases and conditions, including cerebral tissue injury by regulating TLR3/TRIF expression [17, 18, 19, 20]. However, direct evidence of the role of TLR3 in migraine is still lacking. Recently, researchers reported the upregulation of TLR3 in ex vivo brain slices following the induction of cortical spreading depression (CSD) [21], and the modulation of TLR3 by Poly‐IC effectively inhibited CSD‐induced neuroinflammation in rats, which indicates that TLR3 is a critical signaling molecule involved in CSD‐related disorders [22]. Since CSD is associated with several neurological disorders [23, 24], the role of TLR3 and its signaling pathway in migraine still requires further clarification. Therefore, considering the potential involvement of TLR3‐mediated sterile neurogenic inflammation in the pathogenesis of migraine, we aimed to investigate the effects of Poly‐IC on migraine‐associated inflammation and the underlying mechanisms.

In this study, we established a rat model of episodic migraine to explore the beneficial effects of Poly‐IC. We subsequently conducted an in‐depth investigation of the underlying mechanisms, with a focus on inflammatory responses in vitro. These findings could provide better insight into promising new strategies for the treatment of migraine.

## 2 Methods and Materials

All experiments were conducted according to a protocol approved by the Ethics Committee of West China Hospital of Sichuan University and complied with the guidelines outlined in the National Institute of Health's Guide for the Care and Use of Laboratory Animals, as well as the Animal Research: Reporting In Vivo Experiments (ARRIVE) guidelines [25]. The investigators who participated in the behavioral tests and endpoint data collection were blinded to the animal groups. All efforts were made to minimize suffering [26].

### 2.1 Animals

Male Sprague–Dawley rats (weighing 200–220 g, purchased from Chengdu Da‐Shuo Laboratory Animal Co. Ltd.) were housed at the Laboratory Animal Center of West China Hospital of Sichuan University and bred under standard laboratory conditions, including a 12‐h light/dark cycle, a constant temperature, and unrestricted access to food and water.

### 2.2 NTG‐Induced Migraine‐Like Headache Preclinical Model

The NTG‐based migraine model has greatly contributed to the identification of potential mechanisms of migraine pathogenesis and is well suited for conducting repeated controlled experiments [27, 28]. In this study, NTG was purchased from Beijing Yimin Pharmaceutical Co. (Beijing, China) as a fresh dilution of 5 mg/mL in ethanol. We then used 0.9% (*w*/*v*) saline to further dilute NTG to a final concentration of 1 mg/mL. Episodic migraine models were established via a single intraperitoneal injection of NTG at a dose of 10 mg/kg [29]. Rats in the vehicle group were intraperitoneally injected with an equivalent volume of ethanol in saline.

### 2.3 Administration of a TLR3 Agonist and Inhibitor

The immunopotentiator Poly‐IC, a mimic of double‐stranded viral RNA, is known to stimulate TLR3 and is considered an excellent adjuvant in different immunoregulatory models [30, 31]. Poly‐IC obtained from InvivoGen (San Diego, CA, USA) was stored at −20°C until use. Poly‐IC was prepared for injection by resuspending it in sterile saline, mixing it with the solution, heating it to 65°C–70°C at a concentration of 1 mg/mL to ensure complete solubility, and then allowing proper annealing at room temperature. 1 h before NTG injection, the rats were intraperitoneally injected with 0.3 mg/kg Poly‐IC (both the Poly‐IC group and the TLR3/dsRNA complex inhibitor treatment group) [21].

The TLR3/dsRNA complex inhibitor (Calbiochem/Merck) was shown to be a competitive inhibitor of dsRNA in binding to TLR3 with high affinity and specificity [32]. This inhibitor was dissolved in dimethyl sulfoxide, diluted in sterile saline as a working solution, and intraperitoneally administered 1 h before Poly‐IC treatment at 30 mg/kg (TLR3/dsRNA complex inhibitor treatment group) [33]. As a control, rats were treated with an equivalent volume of the appropriate vehicle.

### 2.4 Behavior Test

#### 2.4.1 Tactile Sensitivity Threshold

The mechanical sensitivity threshold of the periorbital region was evaluated with calibrated Von Frey hairs (Semmes–Weinstein monofilaments, Stoelting Co., Wood Dale, Illinois, USA) by applying them to the midline of the forehead and near the eyes using the up–down method [34, 35]. Examples of positive responses included when animals scratched the periorbital region or withdrew their head in response to the stimulus. The tactile thresholds to the stimuli were assessed at baseline and 0.5, 1, 2, 3, and 4 h after NTG or vehicle injection [36].

#### 2.4.2 Head‐Directed Wiping and Scratching

Increased grooming, especially that localized to a specific area (such as the head in migraine models), can indicate increased nociception, as previously described [37, 38]. The head‐directed wiping and scratching results were recorded and calculated after injection.

#### 2.4.3 Rat Grimace Scale

Pain behavior was also assessed based on the Rat Grimace Scale (RGS), the experimental procedures of which were performed as previously described [39]. The changes in orbital tightening and nose‐cheek bulging in each group were recorded after injection according to the RGS.

### 2.5 Primary Neuron Cultures In Vitro

The trigeminal ganglia of neonatal rats were removed and dissociated in 2 mg/mL papain and 1 mg/mL DNase I at 37°C for 30 min for primary neuron culture. A P1000 pipette was used to triturate the digested ganglia. After centrifugation, the cells were resuspended in complete media containing DMEM/F‐12 supplemented with 10% fetal bovine serum and 1% penicillin–streptomycin, plated on precoated 96‐, 24‐, and 6‐well plates, and cultured at 37°C for 4 h after cell counting. The medium was subsequently changed to serum‐free neurobasal‐A medium containing GlutaMax, B27 supplement, and 1% penicillin–streptomycin. The neurons were cultured for 7 days, and on day 3, half the medium was replaced [40, 41].

Capsaicin was used to mimic neurogenic inflammation, which resulted in the release of CGRP from cultured trigeminal ganglion neurons [42, 43, 44]. Briefly, before stimulation, the capsaicin (Sigma, St. Louis, MO, USA) stock solution at a concentration of 100 mmol/L was prepared in dimethylsulfoxide (DMSO, Sigma), diluted in HEPES‐buffered saline (HBSS) and stored at 4°C until use. On the seventh day in vitro, the neurons were washed and incubated with fresh HBSS at 37°C and treated with 2 μM capsaicin for 1 h [42]. Poly‐IC at a concentration of 25 μg/mL was added 24 h before capsaicin stimulation [43, 45]. The TLR3/dsRNA complex inhibitor (100 μM) was then added 1 h before Poly‐IC preconditioning [46]. As a control, the cells were treated with equivalent volumes of the appropriate vehicle.

### 2.6 Vectors and shRNA Transduction

Rat sh*TRIF* and nontargeting control vectors (shControl) were purchased from Genechem. All the viruses express an enhanced green fluorescent protein (EGFP) under the direction of a CMV promoter, which allows the assessment of viral infection.

All the constructs were confirmed via sequencing. The sequence for the control virus was 5′‐TTCTCCGAACGTGTCACGT‐3′, whereas the sequences for sh*TRIF* were 5′‐GGAACGGGCCATAGATCTTGA‐3′ (sh*TRIF#*1) and 5′‐GAGGAAAC CCAATATCAAACT‐3′ (sh*TRIF#*2). Primary neurons were transduced with shControl or sh*TRIF* on the third day in vitro for 72 h before drug stimulation. The multiplicity of infection (MOI) was set as 8.

### 2.7 Western Blot

In vivo, at the end of behavioral testing, trigeminal nucleus caudalis (TNC) segments, which are involved in processing pain sensation in migraine, were rapidly extracted and stored at −80°C. The in vitro neuron cultures were washed with ice‐cold phosphate‐buffered saline (PBS) and digested in 0.125% trypsin at 37°C for 3–4 min. The samples were homogenized in RIPA lysis buffer (CST, USA) supplemented with protease inhibitor (Beyotime, China). The protein concentration was determined using a BCA assay (Epizyme Biomedical Technology Co. Ltd., China). Protein samples were loaded in each lane and were separated via SDS‐PAGE, after which the proteins were transferred onto polyvinylidene difluoride membranes (Millipore, Temecula, CA, USA). After blocking with 5% nonfat dry milk in Tris‐buffered saline containing Tween 20 (TBST) at room temperature for 1 h, the membranes were carefully washed and incubated with primary antibodies against CGRP (Santa Cruz, dilution: 1:50), c‐fos (Abcam, dilution: 1:500), TRIF (Proteintech, dilution: 1:500), TLR3 (Abcam, dilution: 1:500) and β‐actin (Proteintech, dilution: 1:800) at 4°C overnight. The next day, the membranes were carefully washed with TBST and incubated with horseradish peroxidase‐conjugated anti‐mouse or anti‐rabbit secondary antibodies for 1 h at room temperature. The bands were then visualized with an enhanced chemiluminescence kit (Bio‐Rad, USA).

### 2.8 Immunofluorescence Staining

4 h after injection, the rats were deeply anesthetized and perfused with cold PBS (pH 7.4) followed by 4% cold paraformaldehyde (PFA). Regions from the medulla oblongata to the cervical spinal cord (C1–C2) were immediately isolated and postfixed at 4°C for 24 h. The TNC tissue was then sequentially removed, dehydrated, embedded, and cut into 10 μm‐thick transverse sections, which were obtained from representative serial paraffin‐embedded sections and subjected to immunostaining. Cells cultured in vitro were washed with ice‐cold PBS and fixed in paraformaldehyde for 10 min. After three washes in PBS, the cells were permeabilized for 15 min with 0.3% Triton X‐100 in PBS before blocking with goat serum albumin for 1 h at 37°C. Sections/cells were incubated with primary antibodies against TLR3 (Abcam, 1:500), neuronal nuclei NeuN (Abcam, 1:500), GFAP (Abcam, 1:500), Iba‐1 (Abcam, 1:500), CGRP (CST, 1:800), c‐fos (Abcam, 1:200), TRIF (Abcam, 1:500), and MAP‐2 (Invitrogen, 1:10000) overnight at 4°C. Samples were then incubated with fluorescent secondary antibodies for 1 h at room temperature after extensive washing. The nuclei were counterstained with 4,6‐diamidino‐2‐phenylindole (DAPI) for 5 min.

### 2.9 ELISA

In accordance with the manufacturer's instructions, the levels of IL‐6 and TNF‐α secreted by cells in culture and by the TNC were quantified with an ELISA kit (Jiangsu Medical Industrial Co. Ltd.).

### 2.10 Statistical Analysis

The data are presented as the mean ± SEM. The Student's *t* test was used for two‐group comparisons. Differences in the means among multiple groups were analyzed using one‐ or two‐way analysis of variance (ANOVA). Normality tests were conducted prior to performing parametric analyses. Differences were considered significant when *p* < 0.05. GraphPad Prism version 9.0 was used for statistical analyses (GraphPad Software, San Diego, CA, USA).

## 3 Results

### 3.1 Poly‐IC Treatment Alleviates the Hyperalgesia After NTG Injection by Increasing TLR3 Expression

**FIGURE 1:**
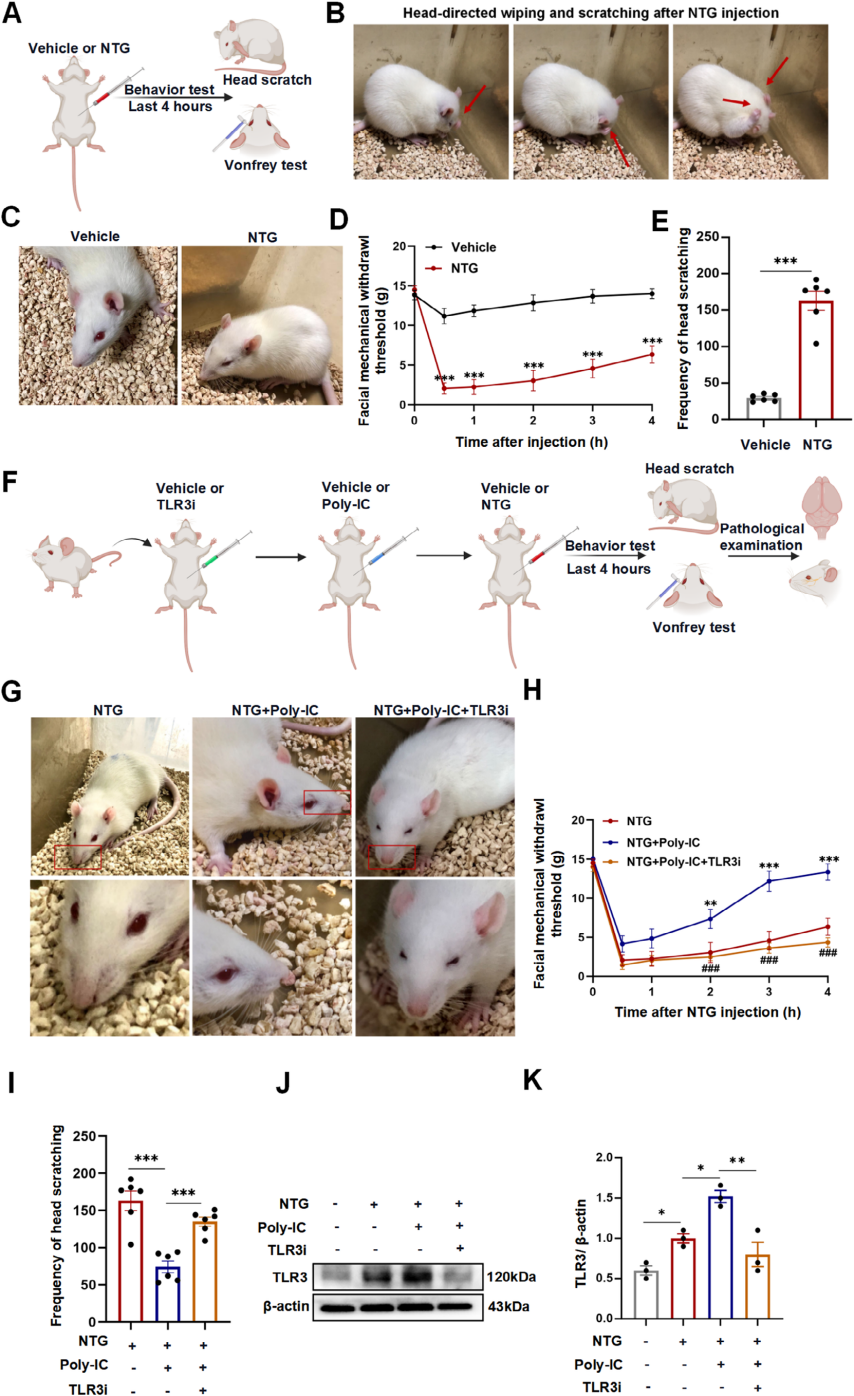
Poly‐IC pretreatment reversed the hyperalgesia induced by NTG injection. (A) Schematic diagram of migraine induction by NTG. (B) Typical behavior of frequent head scratching after NTG injection. (C) In addition to facial signs of discomfort, rats treated with NTG were more languid and exhibited spontaneous eye closure. (D) Mechanical stimulation of the ipsilateral and contralateral orofacial sides after NTG injection measured at different time points. *N* = 6/group. (E) Frequency of head scratching in 2 h after NTG injection. *N* = 6/group. (F) Schematic overview of the experimental design. (G) Several facial signs of discomfort, including partial or complete eye closure, and bulging changes in the cheeks and nose, were observed after NTG injection. Fewer facial signs of discomfort were observed after Poly‐IC treatment. (H) Mechanical stimulation of the ipsilateral and contralateral orofacial sides after Poly‐IC injection. *N* = 6/group. *P represents NTG + Poly‐IC compared with NTG, and ^#^P represents NTG + Poly‐IC compared with NTG + Poly‐IC + TLR3i. (I) Frequency of head scratching within 2 h after Poly‐IC delivery. *N* = 6/group. (J, K) TLR3 expression examined by Western blotting. The data are shown as the means ± SEMs, ***p < 0.001, **p < 0.01, *p < 0.5, ^###^ *p* < 0.001.

The NTG‐induced migraine‐like headache preclinical model is a well‐established experimental method for inducing migraine in rodents. We first investigated migraine‐like hyperalgesia induced by NTG. As shown in the flow chart, male Sprague–Dawley rats were intraperitoneally injected with NTG or vehicle (Figure 1A). The rats exhibited characteristic migraine‐related behaviors, including frequent cephalic grooming and tightly closed eyelids. The typical behavior of frequent head scratching began 3–5 min after NTG administration and occurred more frequently within the first 2 h (Figure 1B,C). In line with these behaviors indicative of discomfort, we detected a decrease in the periorbital sensory withdrawal threshold after NTG delivery, which reached maximal reduction at 60–90 min post injection (Figure 1D). In addition, rats in the NTG group exhibited more frequent head scratching (Figure 1E), which indicated the successful establishment of a migraine rodent model.

Next, we used Poly‐IC, a double‐stranded viral RNA mimic that can stimulate TLR3, together with a specific inhibitor to investigate its effect on migraine‐like hyperalgesia (Figure 1F). Impressively, Poly‐IC treatment significantly induced analgesia in a migraine rodent model. Compared with the NTG group and the Poly‐IC + TLR3 inhibitor group, rats in the Poly‐IC group presented markedly fewer head scratches, higher facial mechanical withdrawal thresholds, and fewer facial signs of discomfort (Figure 1G–I). TLR3 expression was upregulated after NTG stimulation, whereas Poly‐IC‐treated rats presented a more enhanced TLR3 expression with less migraine‐like hyperalgesia (Figure 1J,K). Generally, these results indicate that NTG stimulation may increase TLR3 production during migraine attacks, whereas Poly‐IC can relieve hyperalgesia by further activating TLR3 expression.

### 3.2 NTG Enhances the Expression of CGRP, c‐Fos, and Inflammatory Cytokines, While Poly‐IC Inhibits This Effect

**FIGURE 2:**
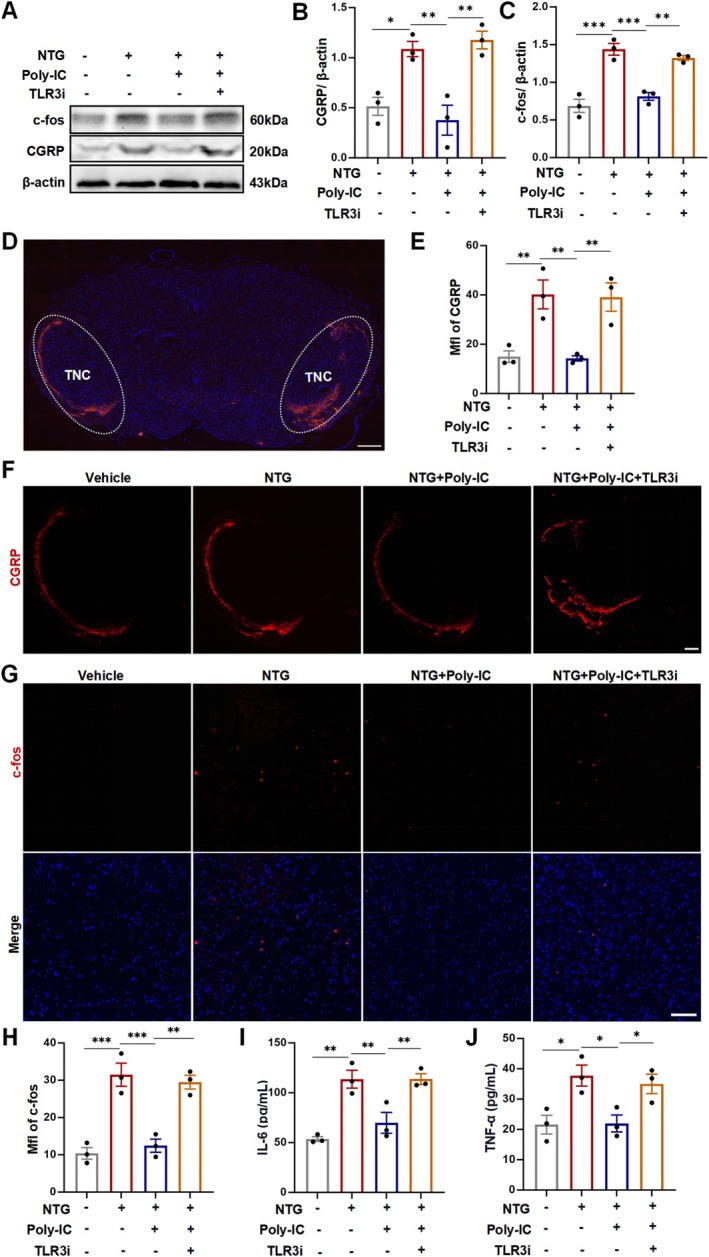
Poly‐IC protected against neurogenic inflammation in a rat model of migraine. (A–C) CGRP and c‐fos expression in TNC tissue examined by Western blotting. *N* = 3/group. (D) Representative image of the TNC area subjected to CGRP immunofluorescence staining. Scale bars = 200 μm. *N* = 3/group. (E, F) Expression of the key neuropeptide CGRP examined by immunofluorescence. Scale bars = 50 μm. *N* = 3/group. (G, H) Immunofluorescence images of c‐fos expression. Scale bars = 100 μm. *N* = 3/group. (I, J) Levels of IL‐6 and TNF‐α in the TNC. *N* = 3/group. The data are shown as the means ± SEMs. ***p < 0.001, **p < 0.01, *p < 0.05.

As described, Poly‐IC pretreatment could relieve NTG‐induced hyperalgesia by increasing TLR3 expression. Given previous reports that implicate the inflammatory response in migraine attacks, we investigated whether Poly‐IC could block the proinflammatory response in migraine. To assess this, we performed Western blotting and immunostaining for CGRP and c‐fos expression in TNC segments [47]. Interestingly, the expression of CGRP and c‐fos was significantly increased after NTG injection, whereas Poly‐IC treatment markedly reversed this phenomenon (Figure 2A–C). Moreover, the average optic density of CGRP‐immunoreactive fibers and the number of c‐fos‐positive cells in the superficial layers of the TNC were both typically reduced by Poly‐IC delivery (Figure 2D–H). Furthermore, NTG stimulation significantly increased the production of IL‐6 and TNF‐α in homogenates from the TNC, whereas Poly‐IC administration markedly reduced the levels of both cytokines (Figure 2I,J). These findings reveal that Poly‐IC treatment can limit inflammation during migraine attacks by further activating TLR3 expression.

### 3.3 Neurons Act as Target Cells for Poly‐IC Treatment in Migraine

**FIGURE 3:**
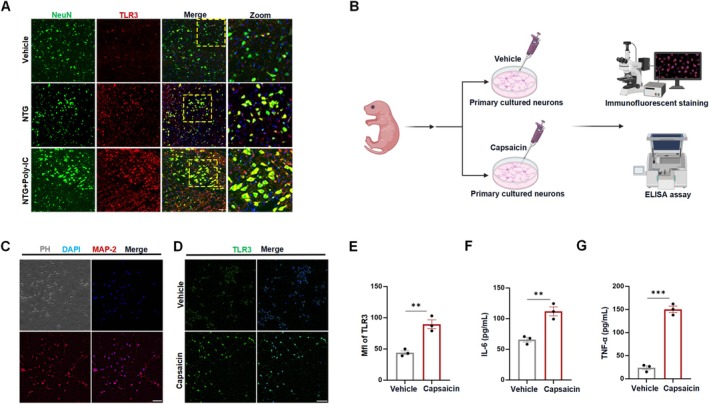
Neurons as target cells for Poly‐IC treatment in migraine. (A) Representative images showing TLR3 expression in neurons after vehicle, NTG, or NTG + Poly‐IC treatment in rats. Scale bar = 50 μm. (B) Schematic diagram of the experimental extraction and stimulation of neurons. (C) Representative bright field and fluorescence images of mature neurons. Scale bars = 100 μm. (D, E) Capsaicin stimulation enhanced TLR3 expression in primary cultured neurons. Scale bars = 50 μm. *N* = 3/group. (F, G) IL‐6 and TNF‐α protein levels in the cell supernatants of cultured neurons after stimulation with capsaicin. *N* = 3/group. The data are shown as the means ± SEMs. ***p < 0.001, **p < 0.01.

**FIGURE 4:**
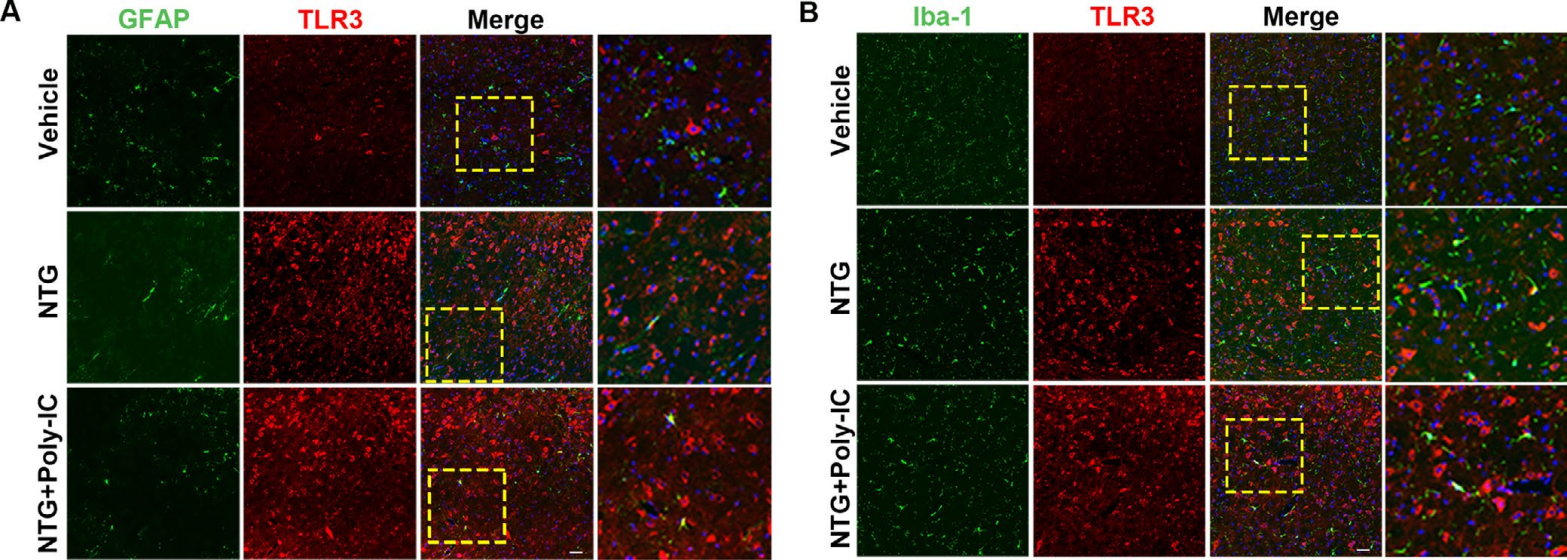
TLR3 expression in different cell fractions. (A, B) Representative images showing TLR3 expression in astrocytes (A) and microglia (B) after vehicle, NTG, and NTG + Poly‐IC treatment in rats. Scale bars = 50 μm.

A remaining objective is to comprehensively understand the cellular and molecular mechanisms by which Poly‐IC protects against neurogenic inflammation in migraine. We performed double immunofluorescence staining to label the TLR3 protein and cell type‐specific markers in the TNC, including NeuN for neurons, Iba‐1 for microglia, and GFAP for astrocytes. Immunostaining revealed that after NTG or NTG + Poly‐IC administration, TLR3 was up‐expression than vehicle group and mainly colocalized with neurons (Figure 3A) rather than microglia or astrocytes (Figure 4A,B) in TNC segments. Therefore, we used primary cultured neurons to further explore the effects of TLR3 signaling in vitro (Figure 3B,C). Primary cultured neurons were treated with capsaicin, the active ingredient of hot chili peppers, which has been used as a chemical stimulus for cultured neurons to mimic neurogenic inflammation, as this compound increases the CGRP concentration [42, 43, 44]. Immunofluorescence staining confirmed that capsaicin stimulation prominently enhanced TLR3 expression in neurons (Figure 3D,E). Moreover, ELISA revealed that the levels of the proinflammatory cytokines IL‐6 and TNF‐α were markedly greater in capsaicin‐treated neurons than in the neurons in the vehicle group (Figure 3F,G). Overall, these results suggest that the NTG‐induced inflammatory response and Poly‐IC effects are neuron‐specific during the pathogenesis of migraine.

### 3.4 Poly‐IC Mitigates Inflammation by Activating the TLR3/TRIF Signaling Pathway

**FIGURE 5:**
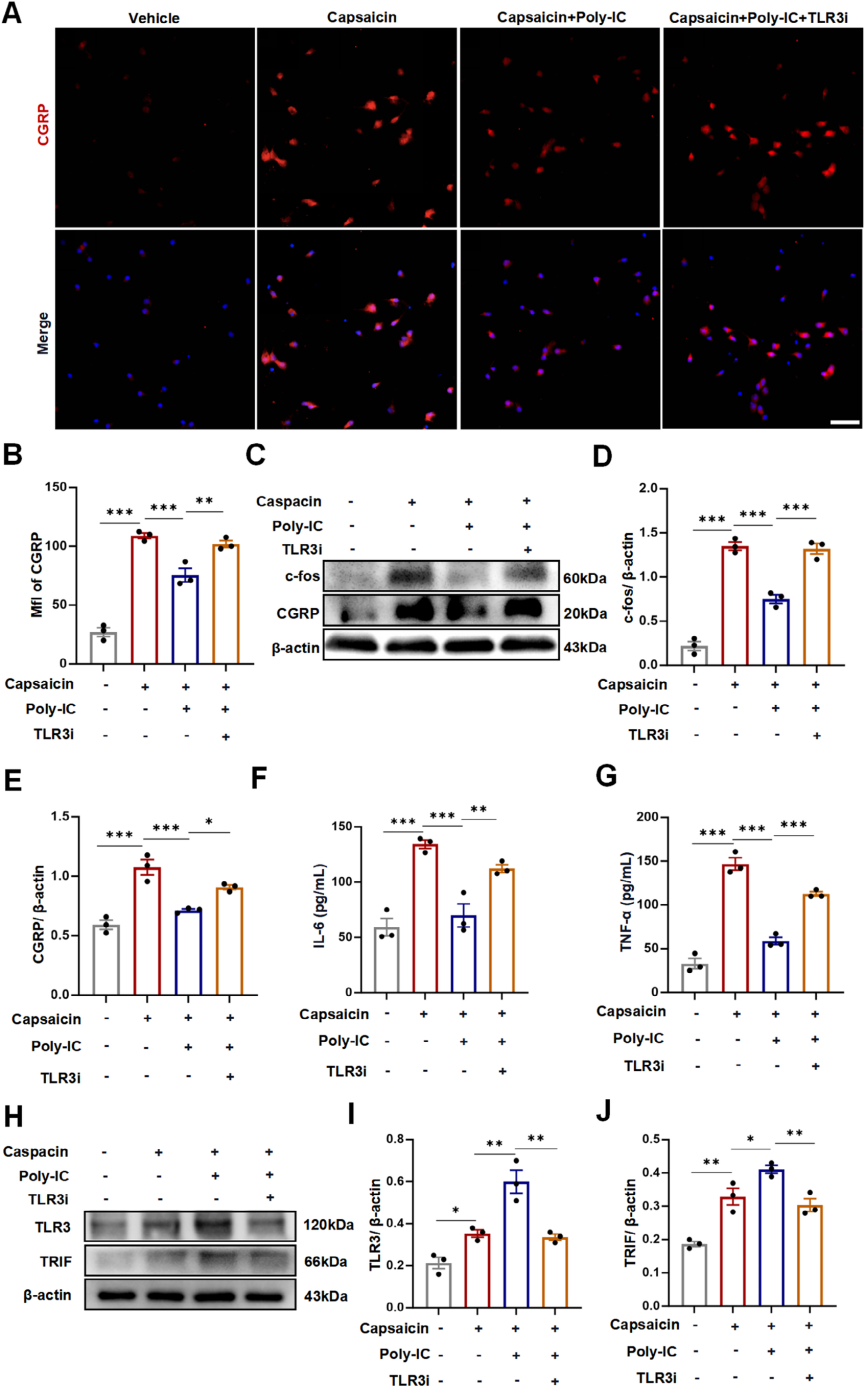
Poly‐IC mitigated neurogenic inflammation in vitro via upregulation of TLR3 and TRIF. (A, B) Expression of CGRP in cultured neurons after stimulation with capsaicin and Poly‐IC pretreatment, as examined by immunofluorescence. Scale bars = 50 μm; *N* = 3/group. (C–E) CGRP and c‐fos expression in neurons examined by Western blotting. *N* = 3/group. (F, G) IL‐6 and TNF‐α protein levels in the cell supernatants of cultured neurons after treatment with Poly‐IC. *N* = 3/group. (H–J) Expression of TLR3 and TRIF in neurons examined by Western blotting. *N* = 3/group. The data are presented as the means ± SEMs. ***p < 0.001, **p < 0.01, *p < 0.05.

We next examined CGRP expression in vitro considering its prominent role in migraine attacks. In accordance with our in vivo observations, the number of CGRP‐positive neurons was significantly increased after capsaicin stimulation, whereas Poly‐IC treatment markedly suppressed CGRP expression in cultured neurons (Figure 5A,B). Furthermore, Western blot analysis revealed pronounced increases in the protein levels of CGRP and c‐fos in primary cultured neurons, whereas Poly‐IC treatment mitigated this phenomenon (Figure 5C–E). In addition, exposure to capsaicin for 24 h notably increased the levels of proinflammatory cytokines, which were reduced in the Poly‐IC group (Figure 5F,G). Furthermore, we determined the involvement of TLR3 signaling in cultured neurons treated with Poly‐IC. Poly‐IC pretreatment significantly increased the production of TLR3 and its adaptor protein TRIF in cultured neurons (Figure 5H–J). These data provide evidence that Poly‐IC treatment inhibits the release of proinflammatory factors via upregulation of TLR3 and TRIF expression.

### 3.5 Poly‐IC Protects Against Neurogenic Inflammation via TLR3/TRIF Signaling Pathway Activation

**FIGURE 6:**
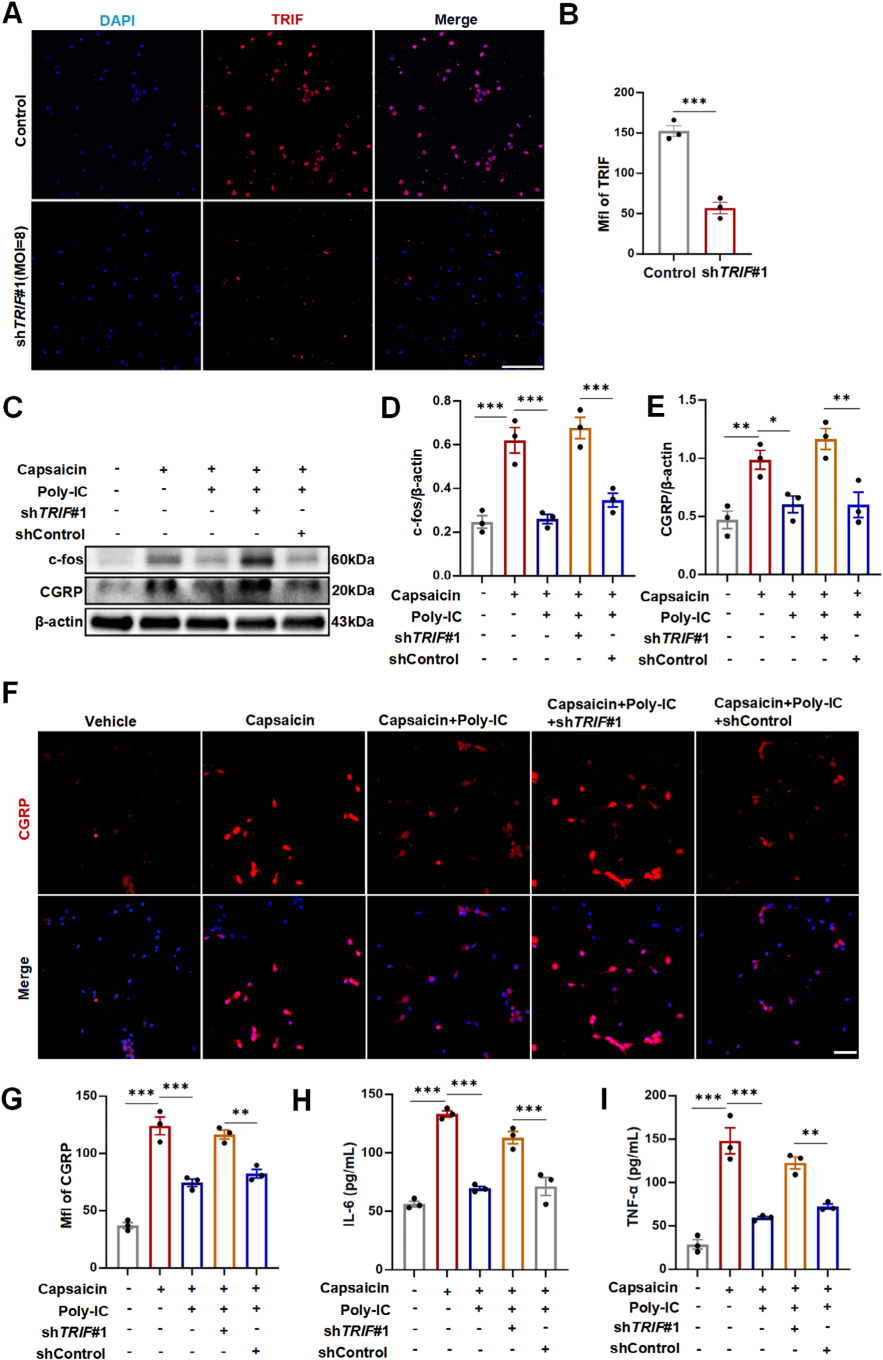
TRIF knockdown reduced the anti‐inflammatory capacity of TLR3 activation in primary cultured neurons. (A, B) Efficacy of TRIF knockdown (MOI of 8 for sh*TRIF#*1) examined by immunofluorescence staining. Scale bars = 100 μm. *N* = 3/group. (C–E) Expression of CGRP and c‐fos after TRIF knockdown examined by Western blotting. *N* = 3/group. (F, G) Expression of CGRP in neurons after TRIF knockdown assessed by immunofluorescence staining. Scale bars = 50 μm. *N* = 3/group. (H‐I) Levels of IL‐6 and TNF‐α secreted by cultured neurons after co‐culture with Poly‐IC and shRNA. *N* = 3/group. The data are shown as the means ± SEMs. ***p < 0.001, **p < 0.01, *p < 0.05.

**FIGURE 7:**
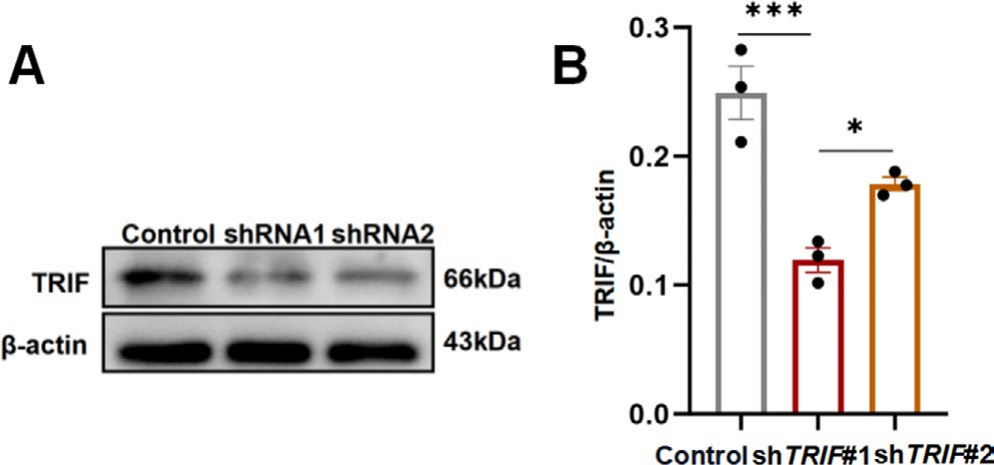
Efficacy of TRIF knockdown by shRNAs in primary cultured neurons. (A, B) Expression of TRIF after shRNA infection examined by Western blotting. *N* = 3/***group. *p* < 0.001, *p < 0.05.

The above results revealed that Poly‐IC treatment activated the TLR3/TRIF signaling pathway and facilitated anti‐inflammatory effects in migraine. To comprehensively understand the functional impact of the TLR3/TRIF signaling pathway on the inflammatory response in migraine, we knocked down TRIF in primary cultured neurons via shRNA. First, Western blotting and immunofluorescence staining confirmed the efficacy of TRIF knockdown at an MOI of 8 for sh*TRIF#*1 (Figures 6A,B and 7). TRIF knockdown induced an increase in CGRP and c‐fos expression in neurons (Figure 6C–E). TRIF knockdown consistently markedly increased the number of CGRP‐positive neurons even under Poly‐IC‐treated conditions (Figure 6F,G), which suggests that reduced TRIF expression weakened the neuroprotective effects of TLR3 activation in migraine. Furthermore, compared with those in the shControl infection group, the levels of IL‐6 and TNF‐α in the cell supernatant from the Poly‐IC with TRIF‐knockdown group were increased (Figure 6H,I). In summary, Poly‐IC protects against neurogenic inflammation via TLR3/TRIF signaling pathway activation in a migraine model. The mechanistic work further revealed that TRIF knockdown reduces the anti‐inflammatory capacity of Poly‐IC treatment in primary cultured neurons.

## 4 Discussion

The present study provides experimental evidence that Poly‐IC protects against neuroinflammation via the TLR3/TRIF signaling pathway in migraine. These results also confirm that TLR3 is involved in migraine attacks. Poly‐IC treatment significantly increased TLR3/TRIF expression, reduced the production of CGRP, c‐fos, and inflammatory cytokines, and alleviated NTG‐induced allodynia. TRIF knockdown weakened the anti‐inflammatory capacity of TLR3 activation induced by Poly‐IC treatment.

The activation of TLR3 can initiate immunomodulatory responses in the brain and is also involved in the pathogenesis of neuropathic pain [17, 18, 19, 20, 48, 49]. To our knowledge, the role of TLR3 in migraine attacks remains unclear, which, to some extent, makes it a “blind spot” of clinical therapies. Recently, Ghaemi and colleagues reported increased expression of TLR3 in ex vivo brain slices after CSD induction, indicating that TLR3 is a critical signaling molecule for CSD‐related neurological disorders [22]. Notably, CSD is involved in several neurological disorders, including migraine aura and stroke [23, 24]. Considering this observation, we established an episodic migraine model via single injection of nitroglycerin into rats and verified that TLR3 is involved in the pathological process of migraine attack. Interestingly, inflammatory stimulation increased TLR3/TRIF production during migraine attacks, whereas further activation of TLR3/TRIF signaling by Poly‐IC relieved hyperalgesia and limited inflammation by increasing TLR3 and TRIF expression. As reported, CSD enhances TLR3 and TLR4 expression in the brain, whereas TLR3 modulation by Poly‐IC inhibits CSD‐induced neuroinflammation in the brain and spleen of rats [21, 22]. Similarly, Yang et al. reported that the activation of TLR3 by Poly‐IC has therapeutic effects against cerebral ischemic injury through downregulation of TLR4 signaling [17]. Since TLR4 is involved in migraine attacks and can be activated in trigeminal ganglion neurons in rodents when endogenous inflammation is induced [50], the balance of activation of these two TLR pathways may contribute to neuroinflammation in the migraine phenotype, but this remains unclear and should be verified in the future. Overall, activation of the TLR3/TRIF signaling pathway, according to our study, is a potential therapeutic strategy to ameliorate proinflammatory activity and disease severity in migraine.

As known, the nociceptive transmission originates from the activation and sensitization of first‐order trigeminal vascular neurons. Injury transmission gradually activates and sensitizes trigeminal vascular neurons in the thalamus, which subsequently transmit pain sensations to cortical areas such as the somatosensory cortex, ultimately leading to migraines. Research has focused on the potential role of glial cells in neuroinflammatory responses; however, more investigations are needed on neurons, which may provide opportunities for further exploration of the pathophysiology of migraine [51, 52]. We performed double immunofluorescence staining to label the TLR3 protein and cell type‐specific markers in TNC and illustrated the pivotal role of neurons in the pathogenesis of migraine, which is in line with these previous findings.

Moreover, pretreatment with the TLR3 agonist Poly‐IC, which activates TLR3 expression in neurons, effectively alleviated pain and reduced neuronal inflammation. Neuroinflammation plays a significant role in most neurological diseases. Additionally, inflammation is generally acknowledged to be critical for migraine pathogenesis. The activation of cortical, subcortical, and brainstem regions and the subsequent release of critical neuropeptides are essential for migraine [53]. As a pleiotropic neuropeptide critically involved in migraine pathophysiology, CGRP induces meningeal blood vessel dilation, plasma extravasation, platelet activation, and mast cell degranulation [54]. When secreted at pathological concentrations, CGRP functions as a pivotal nociceptive mediator that facilitates the initiation, maintenance, and amplification of nociceptive signaling cascades in the trigeminovascular system [55, 56]. Therefore, these neurotransmitters and inflammatory factors have become therapeutic targets for the treatment of migraine [54]. Recent studies have shown that the application of the TLR3 agonist Poly‐IC protects against cerebral ischemic injury by suppressing damaging inflammatory responses and enhancing endogenous neuroprotection [17, 18, 19, 20]. Other researchers have reported that the activation of TLR3 expression reduces TNF‐α levels to attenuate LPS‐induced liver injury [57]. Notably, the current literature provides little evidence of the role of the TLR3‐mediated signaling pathway in migraine pathogenesis. Our investigation provides evidence that Poly‐IC administration induces TLR3 activation, which mechanistically suppresses CGRP secretion and downregulates key proinflammatory mediators (e.g., IL‐6 and TNF‐α) in both animal models and primary neuron cultures. These observations suggest that TLR3 may be a potential therapeutic target for migraine treatment.

A thorough understanding of the mechanisms by which Poly‐IC‐induced TLR3 activation preserves homeostasis in migraine is still needed. Notably, TRIF‐dependent TLR3 signaling is critical for cytokine production in neurons, and neuronal TLR3 activation likely uses TRIF to activate gene expression in innate immunity [58]. As reported, Poly‐IC treatment enhances TRIF protein expression, reduces neuronal apoptosis, and inhibits proinflammatory cytokine production following ischemic injury [17, 18, 19, 20]. Preconditioning with Poly‐IC activates the TLR3 signaling pathway via TRIF, thereby inducing neuroprotection in an experimental stroke model [18]. Moreover, previous studies have shown that innate immune signaling via toll‐like receptor signaling is responsible for persistent pain [59]. In this study, we observed that the activation of the TLR3/TRIF pathway by Poly‐IC treatment can inhibit inflammatory responses and reduce neurogenic inflammation injury in migraine. These findings suggest that TLR3/TRIF signaling plays pivotal roles in inflammatory processing and nociceptive signaling.

Our study has some limitations. This study focused on neuroinflammatory conditions and the subsequent release of proinflammatory cytokines that contribute to the initiation of facilitated pain states, which cannot explain the full mechanism of migraine. We believe the results may underlie the migraine phenotype and introduce a novel drug target with therapeutic potential for migraine attacks. In addition, since the mechanistic work was performed in vitro in our study, more evidence of the involvement of the TLR3/TRIF signaling pathway in migraine attack is needed. Additionally, for the sake of comparability with previous studies, to avoid the influence of hormonal fluctuations on experimental outcomes, we elected to use only male rats to establish an episodic migraine model. The sex differences involved in the pathophysiological mechanisms of migraine therefore require further exploration.

## 5 Conclusions

These findings highlight the critical role of the TLR3/TRIF signaling pathway in migraine, which is associated with neuroprotective and anti‐inflammatory effects after Poly‐IC delivery. As shown, Poly‐IC treatment increased TLR3/TRIF expression, suppressed the production of CGRP, c‐fos, and proinflammatory cytokines, and alleviated migraine‐like hyperalgesia. Therefore, the activation of TLR3/TRIF by Poly‐IC is a promising therapeutic strategy that could be used to counteract migraine attacks.

## Author Contributions

Y.H. and M.M. designed the study, conducted experiments, analyzed the data, and drafted the manuscript. C.L., Y.Z., and Y.L. provided key experimental techniques, helped perform experiments, and analyzed the data. N.C. contributed to the study design. J.F. and L.H. designed the study, supervised the experimental work, and revised this manuscript. All authors read and approved the final manuscript.

## Ethics Statement

The study protocol was approved by the Local Ethics Committee of West China Hospital, Sichuan University.

## Conflicts of Interest

The authors declare no conflicts of interest.

## Supporting information


Data S1.

## Data Availability

The data supporting this study's findings are available from the corresponding author upon request.
