# Supplementary figures and images for: Poly‐IC Alleviates Nitroglycerin‐Induced Migraine by Inhibiting Neuroinflammation via TLR3/TRIF Signaling Pathway
Source: CNS Neurosci Ther. 2025 May 23;31(5):e70444. doi: 10.1111/cns.70444 (PMC12099309; doi:10.1111/cns.70444)

Figure 1J

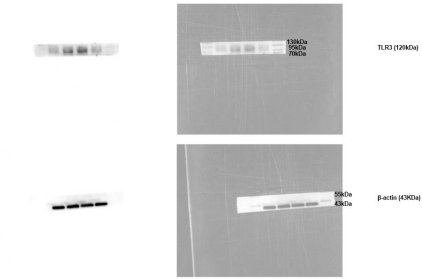

Figure 2A

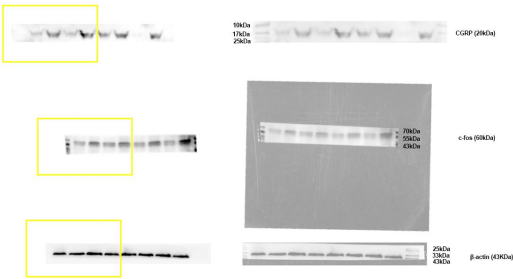

Figure 5C

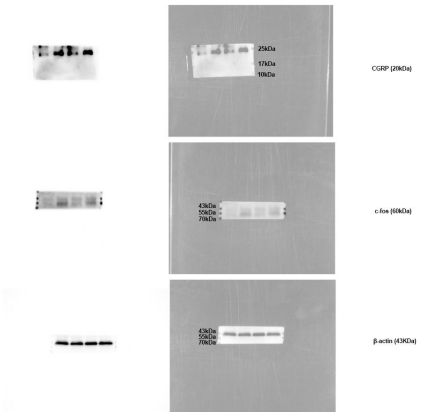

Figure 5H

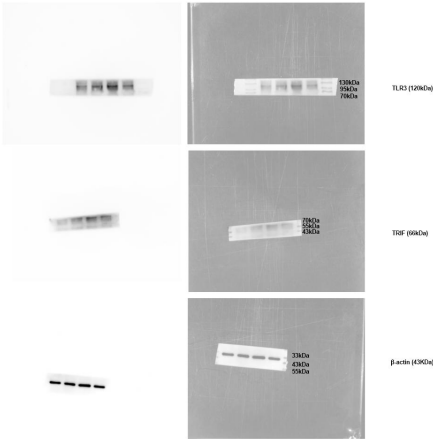

Figure 6C

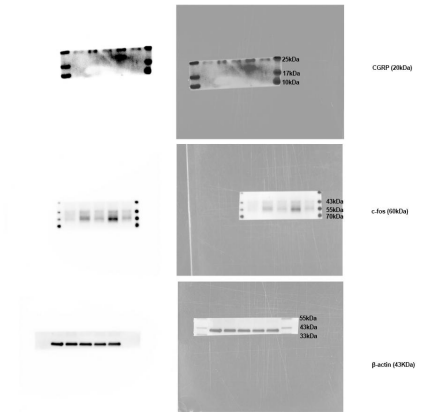

Figure 7

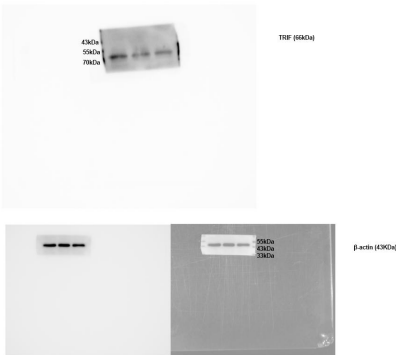

Supplement: Supplementary file 1 — Data S1. [file CNS-31-e70444-s001.pdf]
